# Monitoring Betaine Using Interval Time Between Events Control Chart

**DOI:** 10.3389/fnut.2022.859637

**Published:** 2022-03-31

**Authors:** Muhammad Saleem, Nasrullah Khan, Muhammad Aslam

**Affiliations:** ^1^Department of Industrial Engineering, Faculty of Engineering-Rabigh, King Abdulaziz University, Jeddah, Saudi Arabia; ^2^Section of Statistics, Sub Campus Jhang, University of Veterinary and Animal Sciences, Lahore, Pakistan; ^3^Department of Statistics, Faculty of Science, King Abdulaziz University, Jeddah, Saudi Arabia

**Keywords:** moving average chart, neutrosophic, neutrosophic average run length, Monte Carlo simulation, shift

## Abstract

A generalization of moving average (MA) control chart for the exponential distribution under classical statistics is presented in this article. The designing of the MA control chart for the exponential distribution under neutrosophic statistics is also presented. A Monte Carlo simulation under neutrosophic is introduced and applied to determine the neutrosophic control limits coefficients and neutrosophic average run length and neutrosophic standard deviation for various shifts. The application of the proposed chart is given using Betaine data. The comparison and real example studies show the efficiency of the proposed chart over the existing charts.

## Introduction

Although the Shewhart control charts have been applied widely due to their operational simplicity, these control charts are nevertheless designed and implemented under the assumption that the quality of interest follows the symmetrical distribution. In addition, the Shewhart control charts detect only a big shift in the process. In practice, such as in the chemical process, accelerated life testing and in the healthcare department, the quality of interest does not follow the normal distribution. The Shewhart control charts cannot be applied when the data is skewed, see Derya and Canan (1). Nelson (2) proposed a control chart for the Weibull distribution. Bai and Choi (3) worked on a control chart for skewed data. Zhang et al. (4) proposed a chart for the gamma distribution. Rahali et al. (5) presented a chart for various distributions. More details for such control charts can be seen in Choobineh and Ballard (6). Santiago and Smith (7) used Nelson (8) transformation to convert exponential distribution data to normal and presented the chart to monitor the time between events. Aslam et al. (9) extended Santiago and Smith (7) chart for the repetitive sampling. More details about this type of control charts can be seen in Zhang et al. (4), Aksoy (10), and Borror et al. (11).

The control chart based on moving average (MA), exponentially weighted moving average (EWMA), and cumulative sum (CUSUM) statistics is more sensitive to detecting a small shift in the process. An economic model for the MA chart is introduced by Chen and Yang (12). Wong et al. (13) studied the sensitivity of the MA chart. Khoo and Wong (14) and Areepong (15) proposed an MA chart using a double sampling scheme. Mohsin et al. (16) presented the MA chart using loss function. Alghamdi et al. (17) designed the MA chart for the Weibull distribution.

The fuzzy approach is applied when uncertainty in observations or parameters in presented. According to Khademi and Amirzadeh (18), “fuzzy data exist ubiquitously in the modern manufacturing process.” The fuzzy-based control charts are applied to monitor the process when the data have uncertain observations. Intaramo and Pongpullponsak (19) presented a control chart using the alpha cut approach. Faraz and Moghadam (20) proposed the chart using the fuzzy approach. Zarandi et al. (21) proposed the hybrid chart using fuzzy logic. Faraz et al. (22) proposed the variable chart under an uncertainty setting. Wang and Hryniewicz (23) proposed a fuzzy control chart using the bootstrap approach. Kaya et al. (24) proposed a fuzzy chart for individual observation.

Neutrosophic statistics (NS), which is the extension of classical statistics, works on the idea of neutrosophic numbers. In practice, in our world, the more indeterminate data are obtained than the determinate data; therefore, the use of NS becomes important to deal with such data, see Smarandache (25). The NS can be applied when the data have the neutrosophic numbers. Chen et al. (26, 27) worked on NS and applied in rock engineering. Aslam et al. (28) proposed the Shewhart control charts using NS. Aslam (29) designed the charts for an exponential distribution using NS. More information on NS can be seen in Alhabib et al. (30) and Chutia et al. (31). More applications of the neutrosophic numbers can be seen in Ye (32, 33), Ye et al. (34), Mondal et al. (35, 36), Pramanik and Banerjee (37), and Maiti et al. (38).

By exploring the literature and to the best of our knowledge, there is no work on MA control chart using the exponential distribution under NS. In this article, we use Nelson (8) transformation to propose a chart for the exponential distribution. The neutrosophic Monte Carlo (NMC) will be introduced for the MA chart. We expect that the proposed neutrosophic MA (NMA) chart for neutrosophic exponential distribution (NED) will perform better than the MA chart for neutrosophic distribution under classical statistics. This article is structured as follows: designs of the proposed charts are given in section “Design of the Proposed Charts,” the comparative study is given in section “Comparative Study,” the application of the proposed charts is given in section “Application of Proposed Chart Using Betaine Data,” and some concluding remarks are given in the last section.

## Design of the Proposed Charts

Let the neutrosophic time between event *T*_*N*_ = *T* + *A*_*N*_*I*_*N*_; *I*_*N*_ ∈ [*I*_*L*_,*I*_*U*_], where *T* shows the time between event under classical statistics, *A_N_I_N_* denotes the indeterminate part, and *I*_*N*_ ∈ [*I*_*L*_,*I*_*U*_] denotes the indeterminacy interval follows NED having the neutrosophic scale parameter θ_*N*_ ∈ [θ_*L*_,θ_*U*_]. Aslam (29) introduced the neutrosophic probability density function (NPDF) following form of NED:


(1) $$\text{f}\,\left({\text{t}}_{\text{N}}\right)=\frac{1}{\theta_{\text{N}}}\,{\text{e}}^{-{\text{t}}_{\text{N}}/\theta_{\text{N}}};{\text{t}}_{\text{N}}\geq 0,T_{N}\in \left[T_{L},T_{U}\right],\theta_{\text{N}}\in \left[\theta_{L},\theta_{U}\right]$$

where Γ(*t*_*N*_) denotes the gamma function, see Aslam and Arif (39) for details. The neutrosophic commutative distribution function (NCDF) is given by,


(2) $$\text{P}\left(T_{N}\leq t_{N}\right)+1-\exp\left(-{\text{t}}_{\text{N}}/\theta_{\text{N}}\right);{\text{t}}_{\text{N}}\geq 0,T_{N}\in \left[T_{L},T_{U}\right],\theta_{\text{N}}\in \left[\theta_{L},\theta_{U}\right]$$

The neutrosophic forms of the NPDF and NCDF of NED are written as follows:


(3) $$f\,\left(t_{N}\right)=f\,\left(t\right)+B_{N}\,I_{N};I_{N}\in \left[I_{L},I_{U}\right]$$

and


(4) $$F\,\left(t_{N}\right)=F\,\left(t\right)+C_{N}\,I_{N};I_{N}\in \left[I_{L},I_{U}\right]$$

where *f*(*t*) and *F*(*t*) are PDF and CDF of the exponential distribution under classical statistics. The NPDF and NGD become classical exponential distribution if no indeterminacy is found in the data. According to Nelson (8) and Santiago and Smith (7), if *T*_*N*_ ∈ [*T*_*L*_,*T*_*U*_] follows the NED, then $T_{N}^{*}=T_{N}^{1/\beta_{N}};T_{N}^{*}\in \left[T_{L}^{*},T_{U}^{*}\right]$ follows the neutrosophic Weibull distribution with neutrosophic shape parameter β_*N*_ ∈ [β_*L*_,β_*U*_] and neutrosophic scale parameter $\theta_{N}^{1/\beta_{N}}$. Note here that the neutrosophic Weibull distribution becomes approximately neutrosophic normal distribution when β_*N*_ ∈ [3.6,3.6] having the following neutrosophic mean and variance:


(5) $$\mu_{N}=\theta_{0\,N}^{*}\,\Gamma \,\left(1+\frac{1}{3.6}\right);\theta_{0\,N}^{*}\in \left[\theta_{0\,L}^{*},\theta_{0\,U}^{*}\right]$$

(6) $$\sigma_{N}^{2}=\theta_{0\,N}^{*2}\,\left[\Gamma \,\left(1+\frac{2}{3.6}\right)-\Gamma \,{\left(1+\frac{1}{3.6}\right)}^{2}\right];\theta_{0\,N}^{*}\in \left[\theta_{0\,L}^{*},\theta_{0\,U}^{*}\right]$$

where $\theta_{0\,N}^{*}=\theta_{0\,N}^{1/3.6}$

### Neutrosophic Moving Average Statistic for Exponential Distribution

Suppose that ${\bar{T}}_{1\,N}^{*},{\bar{T}}_{2\,N}^{*},$ be the subgroup averages. The NMA statistic having *w*_*N*_ ∈ [*w*_*L*_,*w*_*U*_] at a time *i* is defined as follows:


(7) $$M\,A_{i\,N}=\frac{{\bar{\text{T}}}_{\text{iN}}^{*}+{\bar{\text{T}}}_{\mathrm{iN}-1}^{*}++{\bar{\text{T}}}_{\mathrm{iN}-w_{N}+1}^{*}}{w_{N}};w_{N}\in \left[w_{L},w_{U}\right]$$

The neutrosophic form of *MA*_*iN*_ ∈ [*MA*_*iL*_,*MA*_*iU*_] can be expressed as,


(8) $$M\,A_{i\,N}=M\,A_{i}+D_{N}\,I_{N};I_{N}\in \left[I_{L},I_{U}\right]$$

Note here that *MA*_*iN*_ ∈ [*MA*_*iL*_,*MA*_*iU*_] reduces to *MA_i_* statistic mentioned in Khoo and Wong (14) when *I_L_* = 0. The neutrosophic mean and variance of *MA*_*iN*_ ∈ [*MA*_*iL*_,*MA*_*iU*_] when *i*≥*w*_*N*_ are given by,


(9) $$E\,\left[M\,A_{i\,N}\right]=\theta_{0\,N}^{*}\,\Gamma \,\left(1+\frac{1}{3.6}\right);M\,A_{i\,N}\in \left[M\,A_{i\,L},M\,A_{i\,U}\right]$$

(10) $$V\,a\,r\,\left[M\,A_{i\,N}\right]=\frac{\theta_{0\,N}^{*2}\,\left[\Gamma \,\left(1+\frac{2}{3.6}\right)-\Gamma^{2}\,\left(1+\frac{1}{3.6}\right)\right]}{n_{N}\,w_{N}};M\,A_{i\,N}\in \left[M\,A_{i\,L},M\,A_{i\,U}\right];n_{N}\in \left[n_{L},n_{U}\right]$$

where *n*_*N*_ ∈ [*n*_*L*_,*n*_*U*_] is the neutrosophic sample size. The neutrosophic upper control limit (NUCL) and neutrosophic lower control limit (NLCL) are given by,


(11) $$U\,C\,L_{N}=\theta_{0\,N}^{*}\,\left\{\Gamma \,\left(1+\frac{1}{3.6}\right)+k_{N}\,\sqrt{\frac{\Gamma \,\left(1+\frac{2}{3.6}\right)-\Gamma^{2}\,\left(1+\frac{1}{3.6}\right)}{n_{N}\,w_{N}}}\right\}$$

(12) $$L\,C\,L_{N}=\theta_{0\,N}^{*}\,\left\{\Gamma \,\left(1+\frac{1}{3.6}\right)-k_{N}\,\sqrt{\frac{\Gamma \,\left(1+\frac{2}{3.6}\right)-\Gamma^{2}\,\left(1+\frac{1}{3.6}\right)}{n_{N}\,w_{N}}}\right\}$$

where *k*_*N*_ ∈ [*k*_*L*_,*k*_*U*_] is the neutrosophic control limits coefficient.

### Neutrosophic Statistic for Exponential Distribution

Suppose that ${\bar{T}}_{1\,N}^{*},{\bar{T}}_{2\,N}^{*},$ be the subgroup averages. The NS having span *w*_*N*_ ∈ [1,1] at a time *i* is defined as follows:


(13) $$M\,A_{i\,N}={\bar{\text{T}}}_{\text{iN}}^{*}+{\bar{\text{T}}}_{\mathrm{iN}-1}^{*}+\cdots +{\bar{\text{T}}}_{\mathrm{iN}-w_{N}+1}^{*};w_{N}\in \left[1,1\right]$$

The neutrosophic form of *MA*_*iN*_ ∈ [*MA*_*iL*_,*MA*_*iU*_] can be expressed as,


(14) $$M\,A_{i\,N\,1}=M\,A_{i\,1}+E_{N}\,I_{N};I_{N}\in \left[I_{L},I_{U}\right]$$

Note here that *MA*_*iN*1_ ∈ [*MA*_*iL*1_,*MA*_*iU*1_] reduces to the traditional X-bar chart mentioned in Montgomery (40) when *I_L_* = 0. The neutrosophic mean and variance of *MA*_*iN*1_ ∈ [*MA*_*iL*1_,*MA*_*iU*1_] when *i*≥*w*_*N*_ are given by,


(15) $$E\,\left[M\,A_{i\,N}\right]=\theta_{0\,N}^{*}\,\Gamma \,\left(1+\frac{1}{3.6}\right);M\,A_{i\,N}\in \left[M\,A_{i\,L},M\,A_{i\,U}\right]$$

(16) $$V\,a\,r\,\left[M\,A_{i\,N}\right]=\frac{\theta_{0\,N}^{*2}\,\left[\Gamma \,\left(1+\frac{2}{3.6}\right)-\Gamma^{2}\,\left(1+\frac{1}{3.6}\right)\right]}{n_{N}};M\,A_{i\,N}\in \left[M\,A_{i\,L},M\,A_{i\,U}\right];n_{N}\in \left[n_{L},n_{U}\right]$$

where *n*_*N*_ ∈ [*n*_*L*_,*n*_*U*_] is the neutrosophic sample size. The NUCL and NLCL are given by,


(17) $$U\,C\,L_{N}=\theta_{0\,N}^{*}\,\left\{\Gamma \,\left(1+\frac{1}{3.6}\right)+k_{N}\,\sqrt{\frac{\Gamma \,\left(1+\frac{2}{3.6}\right)-\Gamma^{2}\,\left(1+\frac{1}{3.6}\right)}{n_{N}\,w_{N}}}\right\}$$

(18) $$L\,C\,L_{N}=\theta_{0\,N}^{*}\,\left\{\Gamma \,\left(1+\frac{1}{3.6}\right)-k_{N}\,\sqrt{\frac{\Gamma \,\left(1+\frac{2}{3.6}\right)-\Gamma^{2}\,\left(1+\frac{1}{3.6}\right)}{n_{N}\,w_{N}}}\right\}$$

where *k*_*N*_ ∈ [*k*_*L*_,*k*_*U*_] is the neutrosophic control limits coefficient.

Suppose that $\text{E}\,\left({\text{M}}_{\mathrm{iN1}}\right)=\theta_{0}^{*}\,{\text{c}}^{1/3.6}.\Gamma \,\left(1+\frac{1}{3.6}\right)$ denotes the shift in the mean of the process. Suppose that neutrosophic average run length (NARL) for the in-control process is *ARL*_0*N*_ ∈ [*ARL*_0*L*_,*ARL*_0*U*_] and for the shifted process is *ARL*_1*N*_ ∈ [*ARL*_1*L*_,*ARL*_1*U*_]. Let *r*_0*N*_ ∈ [*r*_0*L*_,*r*_0*U*_]denotes the pre-defined value of *ARL*_0*N*_ ∈ [*ARL*_0*L*_,*ARL*_0*U*_]. The values of *ARL*_1*N*_ ∈ [*ARL*_1*L*_,*ARL*_1*U*_] in indeterminacy intervals are reported in Tables 1–3 for various values of *w*_*N*_ ∈ [*w*_*L*_,*w*_*U*_] and *n*_*N*_ ∈ [*n*_*L*_,*n*_*U*_]. Table 4 is presented for the neutrosophic control chart for the exponential distribution. The following Monte Carlo simulation under the NS interval method is used to construct Tables 1–4.

1. Draw a random sample *T*_*N*_ = *T* + *A*_*N*_*I*_*N*_;*I*_*N*_ ∈ [*I*_*L*_,*I*_*U*_] of size *n*_*N*_ ∈ [*n*_*L*_,*n*_*U*_] from the NED *f*(*t*_*N*_) = *f*(*t*) + *B*_*N*_*I*_*N*_;*I*_*N*_ ∈ [*I*_*L*_,*I*_*U*_], where *I*_*N*_ ∈ [*I*_*L*_,*I*_*U*_] is variable during the generation of the data.
2. Convert *T*_*N*_ ∈ [*T*_*L*_,*T*_*U*_] to $T{*}_{N}=T_{N}^{1/\beta_{N}};T{*}_{N}\in \left[T{*}_{L},T{*}_{U}\right]$ and compute ${\bar{T}}_{1\,N}^{*},{\bar{T}}_{2\,N}^{*},$ for given subgroups.
3. Compute statistic *MA*_*iN*_ ∈ [*MA*_*iL*_,*MA*_*iU*_] or *MA*_*iN*_ = *MA*_*i*_ + *D*_*N*_*I*_*N*_; *I*_*N*_ ∈ [*I*_*L*_,*I*_*U*_] and plot on *LCL*_*N*_ ∈ [*LCL*_*L*_,*LCL*_*U*_] and *UCL*_*N*_ ∈ [*UCL*_*L*_,*UCL*_*U*_] and record the first out-of-control (run length).
4. Repeat the process 10,000 times and calculate *ARL*_0*N*_ ∈ [*ARL*_0*L*_,*ARL*_0*U*_] and determine the values of *k*_*N*_ ∈ [*k*_*L*_,*k*_*U*_] such that *ARL*_0*N*_ ∈ [*ARL*_0*L*_,*ARL*_0*U*_]≥*r*_0*N*_ ∈ [*r*_0*L*_,*r*_0*U*_]. Determine that values of *k*_*N*_ ∈ [*k*_*L*_,*k*_*U*_] where *ARL*_0*N*_ ∈ [*ARL*_0*L*_,*ARL*_0*U*_] is very close to *r*_0*N*_ ∈ [*r*_0*L*_,*r*_0*U*_].
5. Draw a random sample *T*_*N*_ = *T* + *A*_*N*_*I*_*N*_;*I*_*N*_ ∈ [*I*_*L*_,*I*_*U*_] of size *n*_*N*_ ∈ [*n*_*L*_,*n*_*U*_] from the NED at the shifted mean E(M_*iN1*_).
6. Convert *T*_*N*_ ∈ [*T*_*L*_,*T*_*U*_] to $T{*}_{N}=T_{N}^{1/\beta_{N}};T{*}_{N}\in \left[T{*}_{L},T{*}_{U}\right]$ and compute ${\bar{T}}_{1\,N}^{*},{\bar{T}}_{2\,N}^{*},$ for given subgroups.
7. Compute statistic *MA*_*iN*_ ∈ [*MA*_*iL*_,*MA*_*iU*_] or *MA*_*iN*_ = *MA*_*i*_ + *D*_*N*_*I*_*N*_; *I*_*N*_ ∈ [*I*_*L*_,*I*_*U*_] and plot on *LCL*_*N*_ ∈ [*LCL*_*L*_,*LCL*_*U*_] and *UCL*_*N*_ ∈ [*UCL*_*L*_,*UCL*_*U*_] and record the first out-of-control (run length).
8. Repeat the process 10,000 times and calculate *ARL*_1*N*_ ∈ [*ARL*_1*L*_,*ARL*_1*U*_] using the determined values of *k*_*N*_ ∈ [*k*_*L*_,*k*_*U*_]. Determine that values *ARL*_1*N*_ ∈ [*ARL*_1*L*_,*ARL*_1*U*_] for various shifts *c*.

**TABLE 1:** The values of NARL when *n*_*N*_ ∈ [4,6] and *w*_*N*_ ∈ [3,5].

	k_N_ ∈ [2.738,2.66]		k_N_ ∈ [2.88,2.81]		k_N_ ∈ [2.95,2.89]	
	
c	ARL_0N_ ∈ [200,200]		ARL_0N_ ∈ [300,300]		ARL_0N_ ∈ [370,370]	
0.6	[22.32, 9.54]	[20.58, 6.12664]	[29.82, 10.87]	[27.25, 7.65]	[33.97, 11.79]	[31.99, 8.47]
0.7	[46.44, 19.32]	[45.05, 15.74248]	[65.12, 23.77]	[61.53, 20.21]	[75.85, 27.05]	[74.51, 23.26]
0.8	[96.07, 46.94]	[93.97, 43.6723]	[137.84, 63.56]	[135.3, 60.23]	[171.66, 74.51]	[168.6, 70.46]
0.9	[177.27, 124.57]	[174.87, 119.70458]	[269.35, 183.69]	[263.1, 179.46]	[323.54, 224.7]	[312.74, 218.43]
1	[205.88, 201.75]	[200.64, 198.49039]	[308.15, 305.08]	[300.41, 295.97]	[371.42, 378.18]	[347.9, 359.06]
1.1	[126.66, 102.25]	[124.53, 97.69256]	[183.09, 140.9]	[184.19, 139.73]	[221.82, 170.59]	[220.54, 168.3]
1.25	[50.2, 30.84]	[47.22, 27.46505]	[67.63, 38.43]	[65.02, 34.69]	[78.43, 43.71]	[75.21, 39.74]
1.4	[23.91, 14.54]	[22.29, 11.07722]	[29.73, 17.14]	[27.6, 13.83]	[33.55, 18.25]	[31.97, 14.88]
1.5	[16.57, 10.3]	[15.09, 7.00214]	[20.05, 11.82]	[18.11, 8.61]	[21.98, 12.64]	[19.96, 9.26]
1.6	[12.1, 8.27]	[10.07, 5.0039]	[14.24, 9.02]	[12.55, 5.7]	[15.64, 9.57]	[13.62, 6.22]
1.75	[8.62, 1]	[6.63, 0]	[9.97, 1.01]	[8.19, 0.3]	[10.65, 1.35]	[8.68, 1.73]

**TABLE 2:** The values of NARL when *n*_*N*_ ∈ [6,8] and *w*_*N*_ ∈ [3,5].

	k_N_ ∈ [2.738,2.66]		k_N_ ∈ [2.88,2.81]		k_N_ ∈ [2.95,2.89]	
	
c	ARL_0N_ ∈ [200,200]		ARL_0N_ ∈ [300,300]		ARL_0N_ ∈ [370,370]	
0.6	[12.73, 1.8]	[10.74, 2.5788649]	[15.94, 8.06]	[14.02, 4.7]	[17.77, 8.47]	[15.91, 5.1]
0.7	[28.75, 14.01]	[26.55, 10.7488005]	[38.29, 16.72]	[35.71, 13.18]	[44.12, 18.63]	[41.14, 15.36]
0.8	[66.73, 35.77]	[66.22, 32.0215199]	[94.68, 45.65]	[92.2, 42.26]	[112.74, 54.35]	[109.53, 51.99]
0.9	[154.06, 106.91]	[152.22, 102.9910459]	[218.92, 154.43]	[216.16, 150.4]	[277.03, 185.99]	[273.48, 182.38]
1	[202.36, 196.73]	[197.48, 191.6391117]	[309.28, 295.18]	[294.5, 287.4]	[368.6, 373.67]	[351.58, 350.8]
1.1	[112.4, 89.49]	[109.51, 85.8680966]	[160.01, 123.76]	[156.73, 118.92]	[196.79, 147.58]	[194.22, 144.1]
1.25	[37.31, 24.41]	[35.58, 21.1162176]	[48.86, 30.12]	[46.52, 26.48]	[55.91, 33.44]	[53.9, 29.74]
1.4	[17.29, 11.27]	[15.28, 7.9733226]	[20.73, 12.87]	[18.68, 9.59]	[22.98, 14.11]	[20.74, 10.8]
1.5	[11.63, 8.51]	[9.59, 5.2229717]	[13.67, 9.24]	[11.47, 5.88]	[14.8, 9.77]	[12.7, 6.4]
1.6	[8.62, 1]	[6.85, 0.0806191]	[9.85, 2.35]	[8.08, 3.27]	[10.89, 5.75]	[8.92, 4.75]

**TABLE 3:** The values of NARL when *n*_*N*_ ∈ [8,10] and *w*_*N*_ ∈ [3,5].

	k_N_ ∈ [2.738,2.66]		k_N_ ∈ [2.88,2.81]		k_N_ ∈ [2.95,2.89]	
	
c	ARL_0N_ ∈ [200,200]		ARL_0N_ ∈ [300,300]		ARL_0N_ ∈ [370,370]	
0.7	[19.78, 11.09]	[18.04,7.73952]	[25.38,12.82]	[23.93, 9.61]	[29.59, 14]	[27.02,10.81]
0.8	[50.97, 28.01]	[48.85, 24.77193]	[70.64, 35.46]	[67.77, 32.12]	[82.57, 40.79]	[79.18, 37.17]
0.9	[131, 94.06]	[131.31, 90.24766]	[191.6, 133.08]	[186.54, 130.87]	[238.2, 158]	[234.6 3, 156.44]
1	[200.57, 200.52]	[194.24, 196.53522]	[303.94, 307.75]	[299.06, 296.14]	[363.48, 363.7]	[345.68, 341.27]
1.1	[100.58,80.35]	[99.38, 76.77336]	[143.09, 109.18]	[140.42, 106.71]	[175.19, 129.58]	[175.8, 127.15]
1.25	[29.76, 20.37]	[28.04, 17.08081]	[38.1, 24.26]	[35.33, 21.05]	[43.29, 27.12]	[41.39, 24.09]
1.4	[13.19, 9.61]	[11.3, 6.2903]	[15.76, 10.82]	[14.02, 7.66]	[17.27, 11.37]	[15.13, 8.01]
1.5	[8.92, 1.69]	[7, 2.42547]	[10.49, 7.34]	[8.45, 4.72]	[11.16, 8.2]	[9.44, 4.81]

**TABLE 4:** The values of NARL when *n*_*N*_ ∈ [4,6] and *w*_*N*_ ∈ [1,1].

	k_N_ ∈ [2.79,2.79]		k_N_ ∈ [2.92,2.93]		k_N_ ∈ [2.985,2.985]	
	
c	ARL_0N_ ∈ [200,200]		ARL_0N_ ∈ [300,300]		ARL_0N_ ∈ [370,370]	
0.1	[1.44, 1.04]	[0.8, 0.194473]	[1.64, 1.06]	[1.03, 0.25]	[1.79, 1.08]	[1.19, 0.29]
0.2	[4.17, 1.83]	[3.62, 1.208344]	[5.52, 2.2]	[4.98, 1.65]	[6.41, 2.36]	[5.93, 1.77]
0.3	[10.68, 4.37]	[10.13, 3.794793]	[15.19, 5.79]	[14.61, 5.21]	[18.11, 6.41]	[17.57, 6.01]
0.4	[23.2, 10.4]	[22.55, 10.025824]	[34.06, 14.05]	[33.27, 13.63]	[41.83, 16.37]	[41.17, 16.22]
0.5	[44.84, 22.04]	[43.87, 21.555615]	[67.81, 32.1]	[66.22, 31.73]	[83.76, 37.17]	[83.59, 36.9]
0.6	[81.07, 44.33]	[80.78, 43.679488]	[120.58, 67.85]	[119.43, 67.2]	[150.16, 81.03]	[148.69, 80.57]
0.7	[131.62, 83.46]	[129.61, 83.5705]	[197.56, 131.05]	[195.18, 131.35]	[251.4, 150.61]	[253.75, 148.39]
0.8	[194.52, 146.73]	[191.91, 142.422193]	[301.79, 230.55]	[292.28, 228.68]	[369.5, 274.42]	[358.22, 270.61]
0.9	[240.39, 214.05]	[236.9, 213.334077]	[366.83, 331.58]	[349.68, 320.02]	[445.6, 387.21]	[411.94, 363.41]
1	[215.38, 209.61]	[216.02, 208.398949]	[336.31, 321.66]	[327.1, 310.42]	[399.64, 382.02]	[378.99, 366.89]
1.1	[150.11, 139.4]	[147.34, 137.835766]	[224.51, 206.33]	[227.09, 205.54]	[278.89, 247.58]	[275.79, 243.28]
1.25	[75.6, 60.46]	[75.43, 59.905491]	[104.17, 86.36]	[103.91, 85.21]	[126.16, 99.98]	[127.47, 99]
1.4	[40.31, 29.61]	[39.6, 28.5911]	[53.6, 40.1]	[53.84, 39.43]	[61.81, 44.87]	[61.14, 44.38]
1.5	[28.42, 19.79]	[28, 19.148476]	[36.41, 26.4]	[35.53, 26.17]	[41.94, 29.34]	[41.47, 29]
1.6	[20.29, 14.29]	[19.67, 13.564508]	[25.8, 18.2]	[25.6, 17.78]	[29.19, 19.95]	[28.83, 19.59]
1.75	[13.62, 9.28]	[13.14, 8.847326]	[16.98, 11.46]	[16.53, 10.91]	[18.93, 12.53]	[18.31, 11.79]
2	[8.2, 5.41]	[7.69, 4.90994]	[9.7, 6.42]	[9.23, 5.81]	[10.73, 7.05]	[10.27, 6.53]
2.5	[4.12, 2.86]	[3.6, 2.297476]	[4.74, 3.16]	[4.23, 2.6]	[5.17, 3.34]	[4.67, 2.86]
3	[2.72, 1.99]	[2.17, 1.42443]	[2.99, 2.1]	[2.41, 1.51]	[3.25, 2.22]	[2.75, 1.62]

From Tables 1–4, the following trends are noted in *ARL*_1*N*_ ∈ [*ARL*_1*L*_,*ARL*_1*U*_]:

1. The values of *k*_*N*_ = *k* + *E*_*N*_*I*_*N*_; *I*_*N*_ ∈ [*I*_*L*_,*I*_*U*_] increase when *r*_0*N*_ ∈ [*r*_0*L*_,*r*_0*U*_] increases. For example, when *r*_0*N*_ ∈ [370,370], we note the maximum value of *k*_*N*_ = 2.95−2.89*I*_*N*_; *I*_*N*_ ∈ [0,0.2076] in Table 1.
2. We note the decreasing trend in the indeterminacy interval of *ARL*_1*N*_ = *ARL*_*L*_ + *ARL*_*U*_*I*_*N*_; *I*_*N*_ ∈ [*I*_*L*_,*I*_*U*_] as *n*_*N*_ ∈ [*n*_*L*_,*n*_*U*_] increased and *w*_*N*_ = *w*_*L*_ + *w*_*U*_*I*_*N*_; *I*_*N*_ ∈ [*I*_*L*_,*I*_*U*_] is fixed. For example, when *w*_*N*_ = 3 + 5*I*_*N*_; *I*_*N*_ ∈ [0,0.4] and *c* = 1.5, the value of *ARL*_1*N*_ is *ARL*_1*N*_ = 16.57−10.3*I*_*N*_; *I*_*N*_ ∈ [0,0.6087], which is *ARL*_1*N*_ ∈ [16.57,10.3] when *n*_*N*_ ∈ [4,6]. When *w*_*N*_ = 3 + 5*I*_*N*_; *I*_*N*_ ∈ [0,0.4] and *c* = 1.5, the value of *ARL*_1*N*_ is *ARL*_1*N*_ = 11.63−8.51*I*_*N*_; *I*_*N*_ ∈ [0,0.3666], which is *ARL*_1*N*_ ∈ [11.63,8.51] when *n*_*N*_ ∈ [6,8].
3. We note the decreasing trend of measure of indeterminacy as *n*_*N*_ ∈ [*n*_*L*_,*n*_*U*_] increases.

## Comparative Study

In this section, we compare the performance of the proposed NMA control chart with the proposed neutrosophic control chart for the exponential distribution and control chart under classical statistics in terms of NARL. The proposed NMA control chart is the extension of the proposed neutrosophic control chart for the exponential distribution and control chart for the exponential distribution under NS. The proposed NMA chart reduces to the proposed neutrosophic control chart for the exponential distribution when *w*_*N*_ ∈ [1,1]. Similarly, the proposed NMA chart reduces to Santiago and Smith (7) control chart when *w*_*N*_ ∈ [1,1] and *I*_*L*_ 0. In sections The Proposed NMA Chart vs. Proposed Neutrosophic Control Chart for the Exponential Distribution and “The Proposed Charts vs. Control Chart for the Exponential Distribution Under Classical Statistics”, we present the comparisons of the charts in terms of NARL. In section “Comparisons by Simulation,” we compare the charts using the simulated data.

### The Proposed Neutrosophic MA Chart vs. Proposed Neutrosophic Control Chart for the Exponential Distribution

For a fair comparison between the proposed control charts, we set the same values of the control chart parameters. Tables 1–3 are shown for the proposed NMA chart, and Table 4 presents the proposed neutrosophic control chart for the exponential distribution. By comparing the values of *ARL*_1*N*_ = *ARL*_*L*_ + *ARL*_*U*_*I*_*N*_; *I*_*N*_ ∈ [*I*_*L*_,*I*_*U*_] presented in Table 4 with Table 1, we note that the proposed NMA chart has smaller values of *ARL*_1*N*_ = *ARL*_*L*_ + *ARL*_*U*_*I*_*N*_; *I*_*N*_ ∈ [*I*_*L*_,*I*_*U*_] at all shifts *c*. For example, when *c* = 1.5, the value of the indeterminacy interval of *ARL*_1*N*_ ∈ [*ARL*_1*L*_,*ARL*_1*U*_] is *ARL*_1*N*_ ∈ [19.96,9.26] from the proposed NMA chart. On the other hand, the value of the indeterminacy interval of *ARL*_1*N*_ ∈ [*ARL*_1*L*_,*ARL*_1*U*_] is *ARL*_1*N*_ ∈ [41.47,29] from the proposed NMA chart. From the values of *ARL*_1*N*_ ∈ [*ARL*_1*L*_,*ARL*_1*U*_], it is quite clear that the proposed NMA provides the smaller values of *ARL*_1*N*_ ∈ [*ARL*_1*L*_,*ARL*_1*U*_] as compared to the proposed neutrosophic chart for the exponential distribution. From this comparison, it can be noted that the proposed chart detects a shift in the process between the 9th and 19th samples, whereas the other proposed control chart detects a shift between the 29th and 41st samples. Therefore, the proposed NMA chart is more efficient in detecting the shift in the process as compared to the proposed exponential chart under NS.

### The Proposed Charts vs. Control Chart for the Exponential Distribution Under Classical Statistics

We now compare the efficiency of the proposed control chart under NS with the control chart for the exponential distribution under classical statistics. Note here that the first values of the indeterminacy interval of *ARL*_1*N*_ = *ARL*_*L*_ + *ARL*_*U*_*I*_*N*_;*I*_*N*_ ∈ [*I*_*L*_,*I*_*U*_] in Tables 1–4 represent the average run length (ARL) of the chart under classical statistics. According to the theory of the proposed charts, the proposed charts reduces their competitive chart under classical statistics when *I*_*N*_ ∈ [0,*I*_*U*_]. From Tables 1–4, it is quite obvious that the proposed control charts provide the smaller values of indeterminacy interval of *ARL*_1*N*_ = *ARL*_*L*_ + *ARL*_*U*_*I*_*N*_;*I*_*N*_ ∈ [*I*_*L*_,*I*_*U*_] as compared to the chart proposed by Santiago and Smith (7). For example, when *c* 1.1, the value of ARL from Santiago and Smith (7) chart is 275 and from the proposed chart it is 79. It means, that the existing chart tells about the shift in the process at the 275th sample, whereas the proposed chart tells that the shift can be detected between the 79th and 275th samples. From these comparisons, we conclude that the proposed control charts are flexible, informative, and passable to apply under uncertainty environment.

### Comparisons by Simulation

**FIGURE 1:**
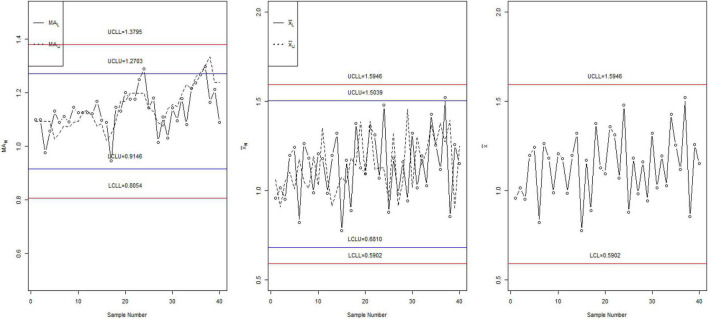
The control charts for simulated data.

To compare the performance of the three charts, we simulated the data from the NED. For the simulation study, let *c* 1.4, *n*_*N*_ ∈ [4,6], and *ARL*_1*N*_ ∈ [370,370]. The first 20 observations are generated from the in-control process, and the next 20 observations are generated from the shifted process when *c* 1.4. The values of NS *MA*_*iN*_ = *MA*_*i*_ + *D*_*N*_*I*_*N*_; *I*_*N*_ ∈ [*I*_*L*_,*I*_*U*_] are calculated and plotted on the three control charts in Figure 1. For these parameters, the tabulated NARL is [33.55, 18.25]. From Figure 1, it can be seen that the proposed control NMA (left in Figure 1) detects the shift at around the 25th sample. The proposed neutrosophic chart for the exponential distribution (middle chart) shows the shift at around the 35th sample, whereas the chart proposed by Santiago and Smith (7) does not show any shift in the process. From this comparison, it can be noted that the proposed chart has the ability to detect the shift in the process early than the proposed neutrosophic chart for the exponential distribution and chart proposed by Santiago and Smith (7).

## Application of Proposed Chart Using Betaine Data

The application of the proposed chart is given using Betaine data. According to Mahmood et al. (41), “Betaine was introduced in artificial rumen containing ruminal fluid of cows. The objective was to determine the rate of disappearance of Betaine from incubation fluid at time points of 0, 1, 2, and 4 h after incubation and feeding of the system.” Note that the first four values are the original data taken from Mahmood et al. (41), and the next 20 observations of the data are generated from the exponential distribution with parameters [0.0042, 0.0076]. The data are shown in Table 5.

**TABLE 5:** The Betaine data.

Sr#	1	2	3	4	Transformed values	MA
1	[260.54, 346.5]	[279.2, 354.39]	[279.4, 357.98]	[294.9, 311.82]	[4.78, 5.06]	[4.16, 3.7]
2	[170.81, 285.4]	[211.33, 289.15]	[217.9, 395.28]	[233.82, 269.9]	[4.4, 4.91]	[4.16, 3.7]
3	[146.27, 203.54]	[158.22, 235.18]	[174.79, 240.81]	[202.12, 312.4]	[4.16, 4.61]	[4.45, 3.7]
4	[73.36, 153.46]	[80.47, 160.16]	[87.2, 162.99]	[40.5, 80.47]	[3.23, 3.91]	[3.93, 3.7]
5	[369.77, 135.99]	[71.36, 280.84]	[6.93, 108.9]	[171.88, 15.94]	[3.58, 3.63]	[3.66, 4.43]
6	[258.31, 225.49]	[25.17, 678.21]	[170.2, 110.81]	[149.87, 90.89]	[3.83, 4.45]	[3.55, 4.3]
7	[108.19, 235.24]	[220.35, 285.29]	[212.79, 80.11]	[89.75, 305.22]	[4.02, 4.41]	[3.81, 4.21]
8	[518.65, 8.64]	[212.35, 57.64]	[271.38, 1.73]	[167.51, 386.99]	[4.75, 2.83]	[4.2, 3.85]
9	[96.44, 54.07]	[3.27, 246.37]	[95.09, 106.38]	[136.92, 20.92]	[3.1, 3.41]	[3.96, 3.75]
10	[827.36, 46.91]	[21.24, 21.08]	[559.93, 165.78]	[113.29, 51.53]	[4.58, 3.09]	[4.14, 3.64]
11	[199.72, 19.1]	[374.88, 12.35]	[488.5, 271.43]	[82.33, 84.31]	[4.63, 3.11]	[4.11, 3.37]
12	[250.81, 514.58]	[182.4, 144.04]	[724.19, 30.82]	[33.88, 241.51]	[4.44, 4.21]	[4.55, 3.33]
13	[71.21, 76.79]	[123.05, 104.74]	[308.2, 49.54]	[479.27, 20.26]	[4.39, 3.06]	[4.49, 3.38]
14	[28.74, 68.61]	[52.5, 185.37]	[402.2, 301.37]	[233.43, 17.8]	[3.85, 3.65]	[4.23, 3.42]
15	[279.76, 36.41]	[107.49, 566.26]	[134.15, 65.84]	[1402.57, 120.33]	[4.96, 3.88]	[4.4, 3.58]
16	[164.63, 244.98]	[101.44, 8.97]	[264.02, 6.6]	[80.55, 29.34]	[3.96, 2.67]	[4.25, 3.49]
17	[60.41, 4.18]	[200.01, 3.19]	[424.25, 35]	[172.37, 173.8]	[4.26, 2.44]	[4.39, 3.14]
18	[798.93, 149.07]	[25.37, 3.67]	[20.11, 151.65]	[1069.05, 20.02]	[4.52, 2.95]	[4.25, 3.12]
19	[102.41, 245.68]	[301.43, 3.75]	[284.25, 105.72]	[139.47, 152.99]	[4.31, 3.44]	[4.36, 3.07]
20	[850.98, 24.59]	[312.86, 22.17]	[182.2, 204.54]	[92.12, 5.64]	[4.8, 2.7]	[4.55, 2.84]
21	[47.36, 35.01]	[120.48, 237.44]	[268.42, 202.57]	[31.73, 26.63]	[3.51, 3.53]	[4.21, 3.01]
22	[7.95, 56.92]	[675.78, 126.87]	[127.54, 66.91]	[308.98, 438.79]	[4.16, 3.89]	[4.16, 3.3]
23	[105.24, 266.52]	[168.57, 398.71]	[12.25, 302.4]	[230.73, 520.94]	[3.58, 5.14]	[3.75, 3.74]
24	[160.21, 127.61]	[82.16, 245.96]	[543.01, 58.72]	[34.75, 73.35]	[3.98, 3.71]	[3.91, 3.79]

**FIGURE 2:**
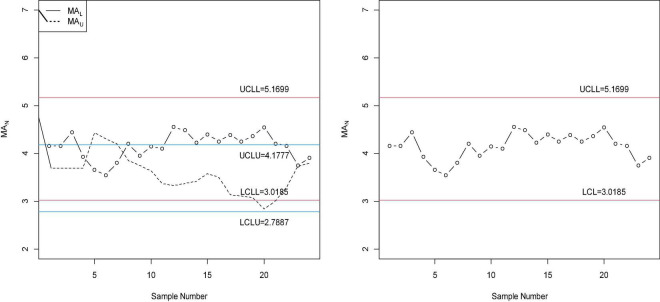
The proposed chart and the existing chart for Betaine data.

The values of NS *MA*_*iN*_ = *MA*_*i*_ + *D*_*N*_*I*_*N*_; *I*_*N*_ ∈ [*I*_*L*_,*I*_*U*_] are plotted on the proposed control chart and on the chart proposed by Santiago and Smith (7) as shown in Figure 2. From Figure 2, it is clear from the proposed control chart that although the control process is normal, some points are near the control limits, which need the Betaine process should be reviewed. On the other hand, using Betaine data, the control chart proposed by Santiago and Smith (7) indicates that the process is in the in-control state, and no action is needed. From this comparison, it is clear that the proposed chart indicates that some values denote indeterminate intervals and need attention.

## Concluding Remarks

A generalization of MA control chart for the exponential distribution under classical statistics is presented in this article. The designing of the MA control chart for the exponential distribution under NS is also presented. A Monte Carlo simulation under neutrosophic is introduced and applied to determine the neutrosophic control limits coefficients and NARL and neutrosophic standard deviation for various shifts. From the simulation study and real example, it is concluded that the proposed chart perform better than the competitors’ control chart in terms of NARL and NSD. The proposed chart is recommended when the practitioner is neutrosophic in sample size or span or observations or all. The proposed control chart using double sampling can be extended as future research.

## Data Availability Statement

The original contributions presented in the study are included in the article/supplementary material, further inquiries can be directed to the corresponding author/s.

## Author Contributions

MS, NK, and MA wrote the manuscript. All authors contributed to the article and approved the submitted version.

## Conflict of Interest

The authors declare that the research was conducted in the absence of any commercial or financial relationships that could be construed as a potential conflict of interest.

## Publisher’s Note

All claims expressed in this article are solely those of the authors and do not necessarily represent those of their affiliated organizations, or those of the publisher, the editors and the reviewers. Any product that may be evaluated in this article, or claim that may be made by its manufacturer, is not guaranteed or endorsed by the publisher.
